# Evaluation of Biochar and Iron as Alternative Materials to Improve Performance of Septic Systems—Part 1. Material Selection and Batch Tests

**DOI:** 10.1002/wer.70207

**Published:** 2025-11-26

**Authors:** Chia‐Yang Chen, Sara Heger, D. Albrey Arrington, Bo Hu

**Affiliations:** ^1^ Water Resources Center University of Minnesota Saint Paul Minnesota USA; ^2^ Loxahatchee River District Jupiter Florida USA; ^3^ Department of Bioproducts and Biosystems Engineering University of Minnesota Saint Paul Minnesota USA

**Keywords:** adsorption, decentralized wastewater treatment, kinetics, nitrogen, phosphorous, wastewater

## Abstract

The benefits of incorporating biochar and iron as alternative materials to improve septic effluent quality were assessed and compared to C33 sand, a traditional material used to construct septic system soil treatment areas. This study used sequential batch tests to investigate pollution reduction performance of C33 sand, eight types of biochar, and three types of iron with various dosages to identify and optimize operational parameters. Pseudo‐first‐order and pseudo‐second‐order kinetics models were used to simulate temporal performance of wastewater treatment and identify likely mechanisms driving improvements in septic effluent quality. Softwood pine (SP) biochar was most effective at reducing biological oxygen demand (BOD), total nitrogen (TN), and fecal coliform (FC) in septic effluent, while treatment with iron‐enhanced‐sand (IES) produced the highest removal efficiency for total suspended solids (TSS) (> 80%) and total phosphorus (TP) (> 95%) among substrates tested. Experimentation revealed dosages that achieved optimal pollutant removal from 50‐mL septic effluent were 5‐g C33 sand, 1‐g SP, or 2‐g IES. Kinetics study showed that the pseudo‐second‐order model generally described the adsorption performance better than the pseudo‐first‐order model regardless of materials (average *R* ^2^ value > 0.95). Furthermore, the pseudo‐second‐order model simulated adsorption capability (mg g^−1^) at equilibrium status with a lower percent error when compared to the pseudo‐first‐order model results. Based on these results, incorporation of SP and IES as alternative materials can achieve higher contaminant removal efficiency and produce cleaner septic effluent, thereby benefiting the environment.

## Summary

- Alternative drainfield materials were tested for septic effluent treatment.
- SP showed superior BOD and nitrogen removal compared to C33 sand.
- IES achieved higher TSS and phosphorus removal than C33 sand.
- Pseudo‐second‐order kinetics model best described temporal uptake dynamics.
- Optimized conditions improved treatment efficiency while reducing cost.

## Graphical abstract

This study quantified pollutant concentrations in septic effluent following treatment with C33 sand, iron products, and biochar materials. These cost‐effective materials offer promising opportunities to reduce pollutant loads in septic system effluent and enhance downstream groundwater and surface water quality.

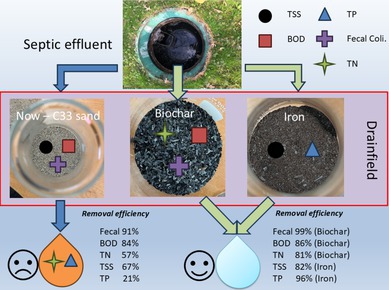

## 1 Introduction

It is estimated that around 20% of buildings in the United States, including single family homes and commercial establishments, use septic systems, that is, onsite sewage treatment and dispersal systems or subsurface sewage treatment systems, to treat over one trillion gallons of wastewater annually (EPA 2002; New et al. 2021). As a result, it is vital that septic systems provide effective wastewater treatment to remove pollutants before effluent is discharged to local groundwater. Typical septic systems are composed of a septic tank, where wastewater treatment occurs through physical processes (i.e., separation of liquid [effluent] from solids [scum and sludge]) and biological processes (i.e., heterotrophic respiration by anaerobic bacteria), and a soil treatment system (i.e., drainfield) that final treatment, including filtration, adsorption, and biological treatment, occurs.

Several studies showed that septic effluent treated with sand was effective at removing contaminants like biological oxygen demand (BOD) and total suspended solids (TSS) (Table S1) (Widrig et al. 1996; Sauer David et al. 1976). Nonetheless, sand filtration of septic effluent was much less effective at removing nitrogen (Dalahmeh et al. 2014), phosphorus (Verma et al. 2019), or bacteria (achieving less than a 2‐log removal of total coliform) (Katukiza et al. 2014), all of which are known pollutants that can degrade surface waters.

Because existing septic systems have the potential to discharge nontrivial loads of nitrogen, phosphorus, and bacteria to local groundwater, numerous total maximum daily load projects have identified septic systems as nonpoint sources of nutrient and bacteria pollution that must be addressed (Benton Soil and Water Conservation District 2013; River 2021). It is important to identify opportunities to improve nutrient and bacteria removal efficiency of existing and new septic systems.

Biochar is a carbon‐rich material (Chen et al. 2020b) produced via oxygen‐limited thermochemical treatments like pyrolysis or torrefaction (Ong et al. 2020). Unlike raw biomass, biochar with higher fixed carbon content (Chen and Chen 2020a; Chen et al. 2021a) possesses properties that make it promising for wastewater treatment through adsorption, due to its high porosity and large surface area (Xiang et al. 2020). Furthermore, biochar can be derived from waste, so using waste‐derived biochar as an adsorbent to treat wastewater supports the circular economy (He et al. 2022) and the waste‐to‐resource concept (i.e., improving wastewater quality [Chen et al. 2021d] while simultaneously mitigating waste production [Chen et al. 2017]). Nonetheless, not all biochar is created equal. Feedstock (Chen et al. 2021b), thermochemical condition (Chen and Chen 2020a), and posttreatment activation can all affect biochar properties, making it critical to identify the biochar most effective for the intended application.

In addition to biochar, iron products have been shown to be effective at removing contaminants, especially phosphorus from water and wastewater. For instance, stormwater treated with iron‐enhanced‐sand (IES) in column tests and field applications showed up to 90% reduction in phosphate concentrations, whereas sand without iron had no capacity to retain phosphate (Erickson et al. 2012). Similarly, the total phosphorus (TP) removal efficiency increased from 64% to 99% when the adsorbent was switched from sand to iron powder (Mei et al. 2020).

Both biochar and iron products can be effective at reducing pollutant loads in wastewater. However, their use in real‐world applications to treat or polish septic effluent remains limited. Most published research has focused on treating synthetic wastewater or stormwater, and the results have been inconsistent. For example, pine wood biochar used to treat brewery wastewater achieved high contaminant reduction rates (94% for chemical oxygen demand, 90% for phosphate, 87% for ammonia, and 82% for TSS) that exceeded the performance of commercial activated carbon (Huggins et al. 2016). Conversely, (Yao et al. 2012) tested 13 biochars and found that more than half exhibited negative removal efficiencies for nitrate and phosphate.

Given the global and local importance of septic systems, this study quantified wastewater quality improvements following treatment with C33 sand, three iron products, and eight biochar materials. The goal of this study was to identify products that could be incorporated into septic system soil treatment areas with the explicit goal of improving effluent quality, that is, decreasing concentrations of TSS, BOD, total nitrogen (TN), TP, and fecal coliform (FC). In addition to evaluating various materials, this study also assessed five dosage rates to optimize pollutant reduction from septic effluent. These findings have the potential to improve groundwater and surface water quality, thereby enhancing downstream public and environmental health.

## 2 Experimental Section

### 2.1 Materials and Wastewater

In this study, 12 materials were assessed for their ability to lower pollutant concentrations in septic effluent: eight biochar materials (1, biochar DG [BD] from The Andersons; 2, biochar pure [BP] from BiocharMerchant.com; 3, naked char [NC] from American Biochar company; 4, softwood pine [SP] from Terra Char; 5, softwood chunk [SC] from Terra Char; 6, hardwood powder [HP] from Terra Char; 7, black ash [BA] from the Natural Resources Research Institute; and 8, red pine [RP] from Natural Resources Research Institute), three iron products (9, IES from Plaisted Companies; 10, zero‐valent iron [ZVI] from Connelly GPM Inc.; and 11, iron tailings [IT] from anonymous company), and 12, C33 sand from Plaisted Companies. C33 sand is a fine aggregate characterized as clean, durable, low in impurities, and uniform particle size distribution. As such, C33 sand is the most commonly used imported material in drainfields and served as the control material in this study. The physical characteristics of all tested materials are shown in Table 1 and Figure S1.

**TABLE 1:** Physical characteristics of the 12 substrates evaluated in this study.

	C33	IES	ZVI	IT	BD	BP	BA	RP	HP	SP	SC	NC
Hydrophobic	—	—	—	—	Yes	Yes	Yes	Yes	Yes	No	No	No
Proximate (%)												
Moisture	~2	< 1	< 1	5.50	4.78	3.15	4.27	1.04	7.07	54.30	64.72	49.23
Ash	~98	> 99	> 99	90.05	7.29	14.56	2.92	2.50	20.92	3.16	5.10	6.87
Volatile matter	< 1	< 1	< 1	4.43	51.04	52.74	56.31	55.48	25.16	31.46	28.30	39.65
Fixed carbon	< 1	< 1	< 1	< 1	36.89	29.55	36.50	40.98	46.84	11.07	1.87	4.24
D10 (mm)	0.13	0.20	0.17	0.71	0.69	0.54	0.57	0.69	0.14	0.39	0.58	0.33
D30 (mm)	0.22	0.35	0.29	1.18	1.29	0.79	0.82	2.06	0.23	0.59	1.15	0.50
D60 (mm)	0.39	0.59	0.47	1.89	2.13	1.66	1.88	2.55	0.37	0.82	2.30	0.77
Cu^a^	2.99	2.91	2.80	2.68	3.08	3.08	3.28	3.67	2.63	2.11	3.95	2.35
Cc	0.95	1.00	1.03	1.05	1.13	0.70	0.63	2.41	0.96	1.10	0.98	0.99
Bulk density (kg m^−3^)	1715	1963	2372	1162	552	184	155	98	326	383	487	242
Particle density (kg m^−3^)	2594	4540	5544	2005	—^b^	—^b^	—^b^	—^b^	—^b^	—^b^	—^b^	—^b^
Porosity (%)	33.88	56.76	57.22	42.03	—	—	—	—	—	—	—	—

Abbreviations: BA, black ash; BD, biochar DG; BP, biochar pure; HP, hardwood powder; IES, iron‐enhanced‐sand; IT, iron tailings; NC, naked char; RP, red pine; SC, softwood chunk; SP, softwood pine; ZVI, zero‐valent iron.

^a^ Coefficient of uniform = D60 / D10; coefficient of curvature = D30^2^ / (D60 × D10).

^b^ Particle density was not analyzed for materials with specific gravity less than that of water (1000 kg m^−3^).

All raw wastewater used in this study was collected from the discharge pipe from a single septic tank located in Le Sueur city in Southern Minnesota. Raw wastewater samples were collected into covered 5‐gal buckets using a peristaltic pump and Teflon tubing. Before pumping samples into buckets, the tubing system was primed and conditioned by pumping septic effluent for 10 min. Once collected, wastewater samples were stored in a walk‐in cooler (4°C). All experiments were completed within 1 week, and wastewater quality did not meaningfully change between Days 1 and 7 (Table 2). Furthermore, to precisely calculate the removal efficiency and adsorption capacity of these adsorbents, a control test without any added adsorbent (i.e., just wastewater in the flask) was conducted to ensure the reduction was not from inherent physical, chemical, or biological reactions. Finally, blank samples (DI water only) were assessed to ensure field and laboratory controls were effective.

**TABLE 2:** Wastewater quality of raw septic effluent.

Raw wastewater (average ± SD)	Day 1 (*n* = 3)	Day 7 (*n* = 3)	Control (*n* = 3)
pH	7.29 ± 0.25	7.22 ± 0.01	7.25 ± 0.01
TSS (mg L^−1^)	56.4 ± 4.88	54.0 ± 5.00	55.0 ± 5.00
BOD (mg L^−1^)	110 ± 28.3	150 ± 45.5	112 ± 7.64
TN* (mg‐N L^−1^)	35.8 ± 3.81	33.8 ± 22.0	36.7 ± 22.1
TKN (mg‐N L^−1^)	35.4 ± 3.82	33.2 ± 22.0	29.6 ± 17.8
NOx^+^ (mg‐N L^−1^)	0.409 ± 0.039	0.563 ± 0.059	7.10 ± 6.30
TP (mg‐P L^−1^)	8.107 ± 0.097	8.35 ± 1.27	8.73 ± 0.81
FC (CFU 100 mL^−1^)	35,500 ± 6123	32,000 ± 19,157	34,667 ± 6128

Abbreviations: BOD, biological oxygen demand; FC, fecal coliform; NOx^+^, nitrate + nitrite; TKN, total Kjeldahl nitrogen; TN*, total nitrogen (=TKN + NOx); TP, total phosphorus; TSS, total suspended solids.

### 2.2 Batch Experiments

The overall methodology of this study is summarized in Table S2. The first batch experiment used C33 sand to form a basis of treatment. Fifteen Erlenmeyer flasks were filled with 50 mL of septic effluent. Then, 0.5, 1.0, 2.0, 5.0, and 10.0 g of C33 sand were added to the flasks (*n* = 3 per treatment). Each flask with septic effluent and C33 sand was placed on an orbital shaker at 120 rpm for 24 h. The experiments were conducted at room temperature (27°C), while the lab temperature was well regulated by a central AC system. After 24 h, the flasks were removed from the orbital shaker and allowed to settle for 15 min. Then, 30 mL of supernatant was collected and analyzed for TSS, BOD, TN, TP, and FC concentrations.

The second and third batch experiments exposed 50 mL of septic effluent to 5.0 g of each biochar material or iron product in Erlenmeyer flasks. Eight biochar materials (i.e., BA, BD, BP, HP, NC, RP, SC, and SP) and three iron products (IES, ZVI, and IT) with three replicates per treatment (e.g., 5.0 g of BA + 50 mL of septic effluent) were assessed. Flasks were placed on an orbital shaker for 24 h, and the same sample collection procedures as in the first batch experiment were followed.

The fourth and fifth batch experiments involved adding five different amounts of SP or IES (0.5, 1.0, 2.0, 5.0, or 10.0 g) to Erlenmeyer flasks containing 50 mL of septic effluent. Three replicates per treatment (e.g., 0.5 g of SP + 50 mL of septic effluent) were placed on an orbital shaker for 24 h, and the sampling procedures followed those used in the previous batch experiments.

### 2.3 Kinetics

To assess temporal dynamics (i.e., kinetics) of pollutant reduction in septic effluent, 1000 mL of septic effluent was exposed to 10, 20, 40, or 100 g of C33 sand; 20 g of SP biochar; or 40 g of IES for 240 min. These effluent‐to‐treatment substrate ratios matched those used in the batch experiments. Three replicates per treatment were placed on an orbital shaker, and supernatant samples were collected in the same manner as previously described at 5, 10, 20, 40, 60, 90, 120, and 240 min and analyzed for TSS, BOD, TN, TP, and FC concentrations. These samples were allowed to settle briefly but not for the full 15 min used in the prior sampling process due to time constraints.

After analysis, the pseudo‐first‐order kinetics model (Equation 1) and the pseudo‐second‐order kinetics model (Equation 2) were used to simulate the kinetic performance and predict equilibrium adsorption capacity.

(1) $$\ln\left(q_{e}-q_{t}\right)=\ln q_{e}-\frac{k_{1}t}{2.303},$$

(2) $$\frac{t}{q_{t}}=\frac{1}{k_{2}q_{e}^{2}}+\frac{t}{q_{e}},$$

where *q*_*e*_ is the maximum adsorption capacity (mg g^−1^) reached after 24 h and *q*_*t*_ is the amount of contaminant adsorbed per gram adsorbent (mg g^−1^) at time *t* (minutes). The rate constants for pseudo‐first‐order and pseudo‐second‐order are *k*_1_ (min^−1^) and *k*_2_ (g mg^−1^ min^−1^). The *q*_*e*_ prediction was calculated using the exponent of the intercept in pseudo‐first‐order kinetics model curve and the inverse of the slope value in pseudo‐second‐order kinetics model curve.

### 2.4 Analysis

Proximate analysis was performed according to ASTM D3172 (ASTM 2013). The moisture content (%) of a sample was reduced by drying the sample at 105°C for an hour, while the ash content (%) was the residual ratio after the thermal treatment at 590°C for 3 h. Volatile matter content (%) was the weight diminished after no‐oxygen thermal treatment (950°C with a 7‐min reaction time), and fixed carbon content (%) was evaluated by the difference between 100 and the sum of moisture, ash, and volatile matter contents. Particle size distribution was performed using a series of mesh sieves. After placing the tested material on the top mesh layer, vigorous shaking was conducted by hand for at least 5 min. The material at each layer was weighed after shaking. To decide the bulk density (kg m^−3^), mass of the tested material was measured after packing it in a graduated cylinder to 100 mL. Particle density (kg m^−3^) was measured by adding a certain mass (~10 g) of tested material into water in a graduated cylinder and recorded the water volume difference. Particle density was not analyzed for materials with specific gravity less than that of water. Porosity (%) was evaluated by the difference between bulk density and particle density.

Wastewater quality analysis followed the Standard Methods for the Examination of Water and Wastewater (Rice et al. 2012) or EPA analytical methods (Keith 1996). Briefly, TSS was calculated based upon the weight difference of a glass fiber filter (Advantec GC‐50) before versus after filtration of a sample. FC analysis was conducted using the filtration method. Vacuum filtration was performed after diluting the wastewater sample with buffer solution (Hach 2143166). The Microdisc membrane filter (MFMCE047045GWS, Membrane Solutions) was placed onto the pad absorbing m‐FC with Rosolic Acid (Hach 2428550) in a petri dish (Fisher Scientific 09‐720‐503) after wastewater filtration. Then, the petri dishes were put upside down in a sealed plastic bag and incubated for 24 h in a water bath at 44.5°C. Buffer solution (Hach 1486266) was prepared for BOD detection, which was calculated as the dissolved oxygen depletion after 5‐day 20°C incubation. Total Kjeldahl nitrogen (TKN), nitrate + nitrite (NOx), and TP analysis were conducted by Hach analytical kits (TNT880, TNT835, TNT 840, TNT844, and DRB200). All wastewater quality analyses were conducted within 2 h after the sample was collected. All values shown in this study were the average number of three replicate samples.

## 3 Results and Discussion

### 3.1 Material Properties

#### 3.1.1 C33 Sand and Iron Products

C33 sand, IES, and ZVI had ash content over 98%, as their major constituents were noncombustible metals (Table 1). IT, a waste generated from the mining process, was somewhat unique among these materials in having ~90% ash content, 5.5% moisture content, and 4.43% volatile matter. For size distribution, the effective sizes (D10) of the first three materials were similar (~0.13–0.20 mm), whereas that of IT was three to five times higher (0.71 mm). Smaller particle size provides higher adsorption capability because of the higher surface area per unit volume (Chen et al. 2015). However, ultrafine particles are more likely to become a source of TSS, which is a contraindication for effective wastewater treatment. The coefficient of uniformity (Cu) (2.68–2.99) and coefficient of curvature (Cc) (0.95–1.05) of these four materials were similar.

ZVI had the highest density (2372 kg m^−3^) and porosity (57.22%) among all the materials tested herein. Larger porosity values indicate larger surface area, which provides better adsorption capability (Dıaz‐Teran et al. 2001). IT had the lowest density as it included the largest fraction of volatile matter among these mineral‐based products. C33 sand had the lowest porosity (33.88%), which physically limits adsorption potential.

#### 3.1.2 Biochars

Variability among the biochars tested likely derived from differences in feedstock and thermochemical conditions during biochar formation (Chen et al. 2021b). NC, SP, and SC were strongly hydrophilic, with moisture contents ranging from 49% to 65%, low volatile matter (28%–40%), and low fixed carbon content (2%–11%) relative to hydrophobic biochars. RP, BP, BA, BD, and HP were hydrophobic, with moisture contents between 1% and 7%. Hydrophobic biochars generally had higher fixed carbon content (30%–47%) and volatile matter (> 50%), except for HP, which had volatile matter content of 25%.

In terms of size distribution, HP had the lowest D10 due to prescreening, whereas SC had the highest, as it was retained on the mesh specified by the manufacturer. Among the remaining biochars, hydrophilic types had smaller particle sizes (0.33–0.39 mm) compared to hydrophobic ones (0.54–0.69 mm), with both types being larger than C33 sand and iron products, except for IT. All materials tested had a Cu less than 4, indicating relatively uniform particle sizes (Figure S1), which facilitates design work that incorporates these materials (Kenney et al. 2013). It is noteworthy that tested biochars were less dense than water, suggesting they may remain suspended in fluid and become a potential source of TSS.

### 3.2 Batch Tests

#### 3.2.1 C33 Sand Dosage Assessment

**FIGURE 1:**
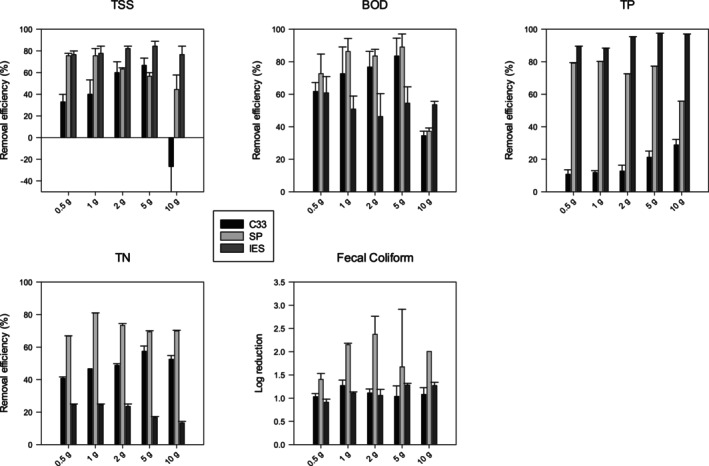
Fifty‐milliliter wastewater beaker test of C33 sand, softwood pine (SP), and iron‐enhanced‐sand (IES) with various dosage amounts (*n* = 3, and error bar represents one standard deviation). BOD, biological oxygen demand; TN, total nitrogen; TP, total phosphorus; TSS, total suspended solids.

To set up a baseline for the entire research, 50 mL of septic effluent was exposed to varying amounts of C33 sand for 24 h. The results (Figure 1) showed that the removal efficiency of most contaminants increased with increasing dosage of C33 sand, except at the highest dosage (10 g per 50‐mL septic effluent), which resulted in negative TSS removal efficiency (i.e., TSS increased compared to the concentration in raw septic effluent) (Figure S2). More C33 sand provided more adsorption sites, which facilitated adsorption and yielded higher removal efficiency (Chen et al. 2021c). Nonetheless, at the highest dosage, the increase in TSS was likely due to fine particles present in the C33 sand used in this study, which had the smallest effective particle size among all materials tested (Table 1). Furthermore, ASTM standards for C33 sand allow up to 3% by weight of clay lumps and friable particles and up to 3% of material finer than 75 μm. These very fine, hard‐to‐settle particles likely contributed to elevated TSS in samples with the highest C33 sand dosage. These samples also showed reduced BOD and TN removal efficiencies, with the greatest removal of BOD and TN observed at the 5‐g C33 sand per 50‐mL septic effluent dosage.

C33 sand reduced TP concentrations by 30% or less, consistent with previous studies indicating that sand was not effective at removing significant amounts of phosphorus from various types of wastewater (Table S1). C33 sand achieved 1–1.3 log reduction in FC, and the removal was not obviously affected by C33 sand dosage. Considering all the wastewater quality parameters assessed, 5‐g C33 sand per 50‐mL septic effluent dosage was identified as optimal for achieving the greatest removal efficiency.

Overall, C33 sand provided effective treatment through physical adsorption, driven by its small particle size and large surface area, which facilitated the removal of TSS, BOD, and FC. However, its chemical inertness limits its ability to remove phosphorus. C33 sand is a silica‐based material, which lacks active mineral components such as iron, calcium, or aluminum that are known to facilitate phosphate uptake (Hinsinger 2001). The zeta potential of silica under neutral pH is generally negative (Muneer et al. 2022; Vitorge et al. 2013), which cannot process the electrostatic attraction properly as the phosphate species under neutral pH are H_2_PO_4_^−^ and HPO_4_^2−^ (Corson et al. 2024). These factors contribute to the minimal phosphorus removal efficiency of C33 sand and support findings from other studies, which reported adsorption capacities of only 10 μg g^−1^ sand and phosphorus broke through pilot‐scale sand filter tanks within the first week (Fischer 1999).

#### 3.2.2 Biochar Screening Tests

**FIGURE 2:**
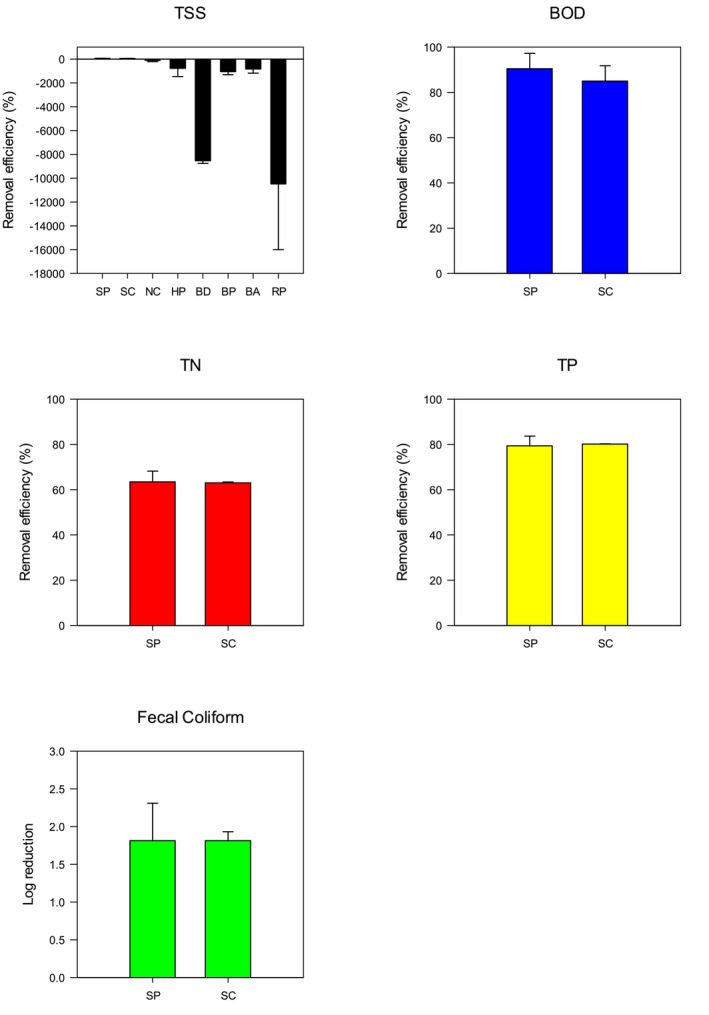
Biochar screening test using the dosage amount of 5 g 50 mL^−1^. BA, black ash; BD, biochar DG; BOD, biological oxygen demand; BP, biochar pure; HP, hardwood powder; NC, naked char; RP, red pine; SC, softwood chunk; SP, softwood pine; TN, total nitrogen; TP, total phosphorus; TSS, total suspended solids.

Septic effluent was then exposed to 5 g of eight different biochar materials to determine their capacity to improve effluent quality and identify which biochar material provided optimal wastewater quality improvement. Most biochar materials showed clear signs of disintegration, that is, water turned black in color (Figure S2), after shaking for 24 h at 120 rpm. During the beaker test, biochar was broken down into very fine particles, which likely contributed to increased TSS. Consequently, negative TSS removal efficiencies were observed in most biochar trials, reaching up to −10,000%, except for SP and SC (Figure 2). Previous studies have also reported that biochar can physically disintegrate, especially after water sorption (Spokas et al. 2014). Thermochemical treatment is known to enhance the grindability of biomass (Correia et al. 2017), meaning biochar becomes more brittle and less structurally durable due to the breakdown of biomass structure during processing. This is likely to explain the widespread disintegration observed after 24 h of shaking.

To quantify this disintegration, size distribution tests were performed on the spent biochar, using the percentage of biochar mass ≤ 0.5 mm (denoted as AP0.5) as an objective metric. All raw biochars had AP0.5 values below 30%, except for HP (85%), which was a powder‐type biochar. After disintegration, AP0.5 values ranged from 14% to 97%, with six out of eight biochars exceeding 30%, providing clear evidence of disintegration. Biochar types that showed significant disintegration were excluded from further analysis in this study, leaving only SP and SC for additional assessments.

SP had higher BOD removal efficiency (90.30% ± 5.57%) compared to SC (84.67% ± 5.59%). SP had a smaller effective particle size (Table 1) and a larger surface area per unit volume that likely increased the possibility of contacting contaminants and performed adsorption. For the other pollutants, the removal efficiencies between SP and SC were within 2%, and the differences were not statistically significant. Therefore, SP was identified as the most effective biochar and was selected for subsequent experiments.

#### 3.2.3 Iron Materials Screening Tests

**FIGURE 3:**
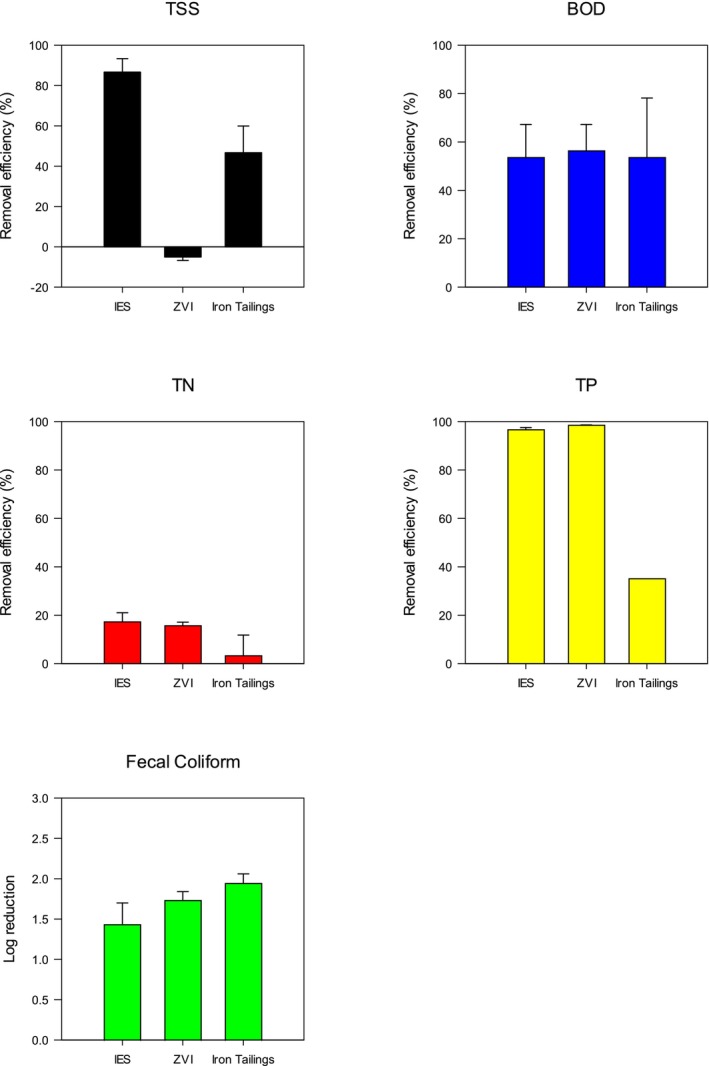
Iron products (iron‐enhanced‐sand [IES], zero‐valent iron [ZVI], and iron tailings) screening test using the dosage amount of 5 g 50 mL^−1^. BOD, biological oxygen demand; TN, total nitrogen; TP, total phosphorus; TSS, total suspended solids.

Similarly, 50 mL of septic effluent was exposed to three iron products (IES, ZVI, and IT) for 24 h, and wastewater quality improvements were assessed. After 24 h of exposure to ZVI, septic effluent TSS increased (i.e., negative TSS removal efficiency), whereas IES achieved the highest TSS removal efficiency (86.67%) among all materials tested (Figure 3). Iron is known for its high surface area (surface‐to‐volume ratio), making it a highly effective adsorbent (Tao et al. 2023). Because adsorption is the primary mechanism for TSS removal, it is not surprising that IES performed best in this regard. In contrast, ZVI appeared to oxidize into ferric oxide during the 24‐h reaction period. Ferric oxide is insoluble in neutral water and, if present as fine particles, may contribute to TSS due to poor settling. The color change in the water confirmed the presence of ferric oxide (Figure S2).

The addition of iron materials produced negligible improvements for TN concentrations (3%–18%) and moderate improvements for BOD (53%–57%). However, the highest TP removal efficiencies among all tested materials were achieved by IES and ZVI (~98%). Iron is known to react with and precipitate phosphorus (Peng et al. 2021). The lowest TP removal efficiency among the iron substrates was observed for IT (< 40%), which was the byproduct of ore processing and much of the iron was in a stable mineral form (e.g., hematite) that did not provide reactive surface sites for phosphorus binding. FC removal efficiencies ranged from 96% to 99% across all iron materials. Based on its superior TSS and TP removal performance and considering that iron addition is primarily targeted for improving these parameters, IES was selected as the optimal iron material for further testing in this study.

#### 3.2.4 SP Biochar Dosage Assessment

To identify the optimal amount of SP biochar to use in benchtop studies treating septic effluent, 50 mL of septic effluent was exposed to 0.5, 1, 2, 5, or 10 g of SP biochar for 24 h, following the same protocol used for C33 sand (see Section 3.2.1 and Table S2). For SP, the results showed an inverse trend in TSS removal efficiency compared to C33 sand (Figure 1), with the greatest TSS removal observed at the lowest SP dosage. As discussed previously, biochar disintegrated in septic effluent. Although SP provided positive TSS removal efficiency at higher dosages, disintegration of SP deteriorated the TSS reduction performance.

For BOD, the 1‐, 2‐, and 5‐g SP dosages resulted in high BOD removal efficiencies (83.64%–89.09%), whereas 0.5 g was moderately effective (73%), and 10 g of SP was the least effective (32%). These results suggest a trade‐off between adsorption (treatment) and disintegration (contamination): Lower dosages may lack sufficient adsorption sites, while higher dosages may introduce excessive fine particles that interfere with treatment. For TN, the 1‐g SP dosage provided superior removal efficiency relative to the other dosages tested. For TP, both 0.5‐ and 1‐g SP dosages showed similar performance, but only the 1‐g dosage achieved 80% removal efficiency. FC removal was the greatest at 2 g (nearly 2.3 log), followed by 1 g (2.15 log) and then 10 g, with high variability at 5 g, and the lowest removal at 0.5 g (< 1.5 log). Taking all these factors together, 1 g of SP biochar per 50 mL of septic effluent was selected as the optimal dosage for future studies.

Overall, SP demonstrated significantly greater nutrient reduction capability in comparison with C33 sand. Under neutral conditions (pH = 6–8), the zeta potential of biochar (−30 to −60 mV) (Yuan and Xu 2011) is lower than that of sand (−10 to −40 mV) (Vitorge et al. 2013), making biochar more effective at ammonia adsorption via electrostatic attraction. Because over 90% of nitrogen in septic effluent was ammonia (Table 2), this stronger adsorption capacity likely contributed to SP's ~30% higher TN removal efficiency compared to C33 sand.

Regarding removal efficiency of TP by SP, it is widely reported that metals including calcium and magnesium (Adhikari et al. 2024; Nobaharan et al. 2021) are present in biochar because they are essential elements in plants, which are the feedstock for many biochars (Kamali et al. 2021). These metals exhibit a strong affinity to phosphorus through both hydrogen bonding and precipitation reactions (Wang et al. 2022), enhancing TP removal efficiency. The improvement of BOD and FC removal efficiency by using SP stems from its high surface area that facilitates adsorption (Singh et al. 2021) as well as its functional groups that are conducive to oxidation processes (Sun et al. 2024). Several studies have shown that the functional groups on biochar surface can facilitate advanced oxidation processes (AOP) by generating OH radicals (Sun et al. 2024; Zhang et al. 2023), and AOP is a well‐documented method to degrade both BOD and FC.

#### 3.2.5 IES Iron Dosage Assessment

The results of dosing septic effluent with five different amounts of IES are shown in Figure 1. IES was clearly the best adsorbent for removing TSS, achieving the highest removal efficiency among all materials tested when 5‐g IES was added to 50 mL of septic effluent. Similar to the trade‐off observed in the SP case, TSS removal was competing with ferric oxide formation as described in the beaker test (Figure S2), which resulted in decreased TSS removal efficiency at the highest IES dosage.

IES gave the lowest removal efficiencies among tested materials for BOD (46%–61%) and TN (13%–25%), consistent with results shown in Figure 3. IES is produced using ZVI, which acts as a reducing agent in water (Li et al. 2024), which is the opposite of what is needed (i.e., oxidation) to remove organic matter (BOD) and TKN (95% of TN in raw wastewater) from septic effluent. Furthermore, under neutral conditions (pH = 6–8), unlike C33 sand and SP, the zeta potential of iron is generally positive (Meng et al. 2016; Shen et al. 2022; Zhou et al. 2020), which inhibits electrostatic attraction with ammonia (which constitutes ~90% of influent nitrogen), resulting in poor TN removal. Notably, the TN removal efficiencies observed in this study (SP > C33 > IES) were inversely correlated with the zeta potential of the tested materials (IES > C33 > SP), suggesting that electrostatic attraction was the primary mechanism driving TN removal.

IES produced the greatest TP removal efficiency (88%–95%) among all materials tested. For the removal efficiency of FC, there was no significant difference between IES (0.9–1.3) and C33 sand (1–1.3). IES consists of 5% ZVI and 95% C33 sand according to the manufacturer's specifications. If there is no specific removal mechanism provided by iron, the observed FC removal efficiency for IES should be similar to that of C33 sand, which is what was observed. Based on these findings, IES is a promising alternative material for incorporation into septic systems due to its ability to significantly reduce TSS and TP concentrations in septic effluent. Although 5‐g IES yielded the highest removal efficiencies for TSS and TP, the difference between 5 and 2 g was not statistically significant. Therefore, 2‐g IES was recommended for future studies, as it offers comparable performance with greater cost‐effectiveness.

**FIGURE 4:**
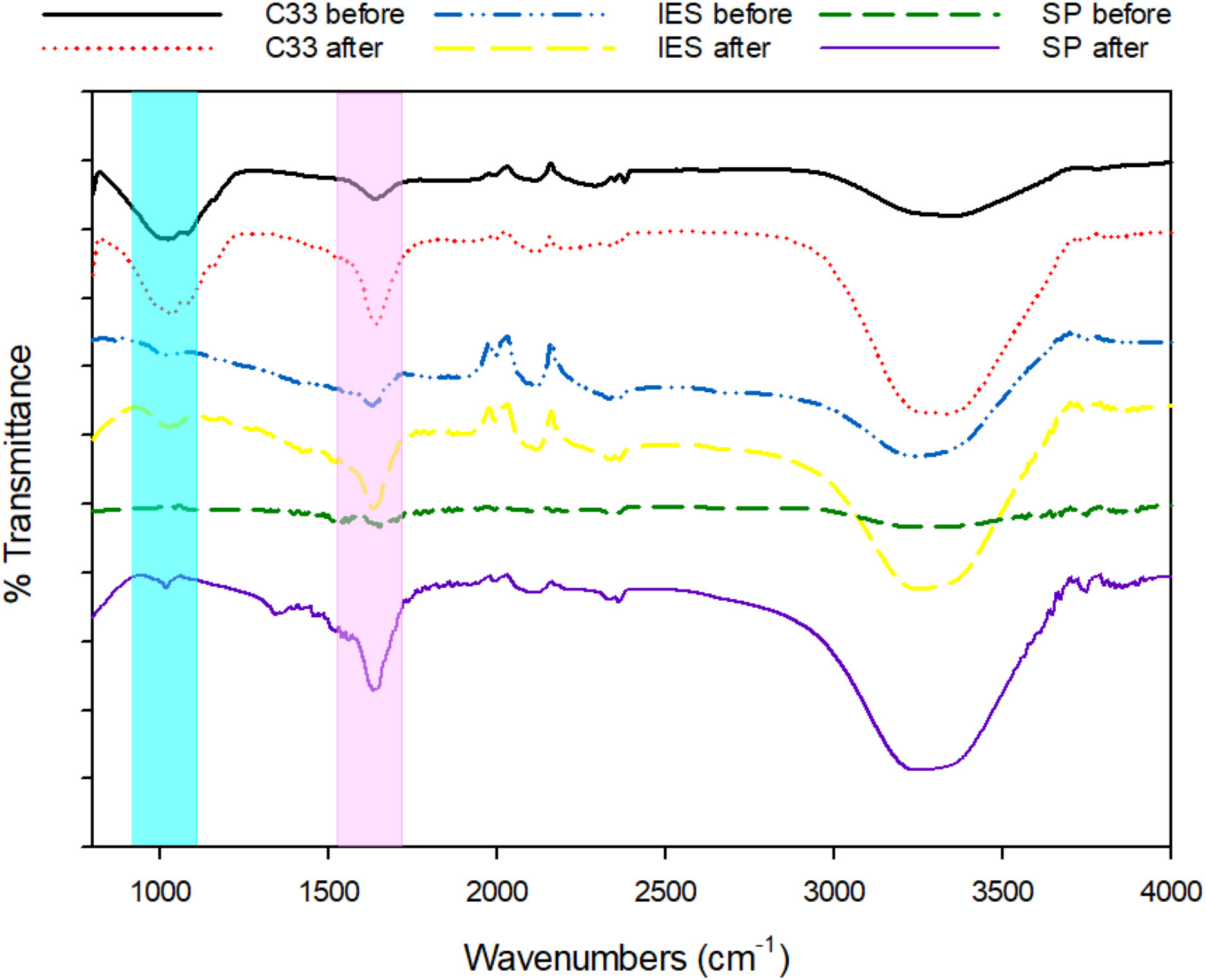
FTIR diagram of C33 sand, IES (iron‐enhanced‐sand), and SP (softwood pine) before and after adsorption test. The designed wavenumbers are (1) 900–1100 cm^−1^ as phosphorus (light blue) and (2) 1580–1650 cm^−1^ as nitrogen (pink).

To support the proposed adsorption/precipitation mechanism, Fourier transform infrared spectroscopy (FTIR; JASCO 6800) coupled with Attenuated Total Reflectance was employed to examine the functional groups on the surface of adsorbents (Figure 4). C33 sand (5 g), SP (1 g), and IES (2 g) were analyzed before and after beaker tests to elucidate the differences in surface chemistry.

Although the N–H stretching region (~3200–3400 cm^−1^) overlaps significantly with O–H vibrations (mainly from adsorbed moisture), making it difficult to attribute changes exclusively to nitrogen, the band observed between 1580 and 1650 cm^−1^, corresponding to N–H bending vibrations (Coates 2000), remained distinct and free from spectral interference. This band was therefore used as an indicator of nitrogen‐containing species on the adsorbent surface. Similarly, the region between 900 and 1100 cm^−1^, attributed to P–O stretching vibrations, was used to represent surface‐bound phosphorus species.

Postadsorption spectra revealed that the most pronounced increase in the N–H bending peak occurred in the SP sample, followed by C33 sand, with the IES exhibiting the least change. This trend aligned well with the nitrogen removal efficiencies observed in the beaker tests (Figure 1), thereby corroborating the adsorption capacity for nitrogen. Additionally, a distinct band appeared around 2100–2200 cm^−1^ after adsorption, attributed to C≡N or other nitrogenous species (Coates 2000), further supporting nitrogen retention on the SP surface.

Regarding phosphorus, the peak difference between spent and virgin material was the most prominent for IES, proving its strong affinity for phosphate. Although SP also showed a noticeable increase in this region after adsorption, the change was less pronounced than that of IES. In contrast, the C33 sand sample exhibited minimal difference before and after adsorption. These observations closely mirrored the phosphorus removal efficiencies obtained from the beaker experiments, where the trend followed IES (95.51%) > SP (80.15%) > C33 (21.33%).

#### 3.2.6 Kinetics

The kinetic study results for C33 sand at various dosages are presented in Figures S3–S7 and Table 3. The *q*_*t*_ of TSS increased with reaction time, reaching saturation at approximately 2 h for the lower dosage and 4 h for the higher dosage (Figure S3). In these cases, longer reaction times permitted more adsorption reactions to occur, saturating the available adsorption sites on the adsorbent. The lower dosage exhibited a higher uptake capacity compared to the higher dosage, consistent with findings from a previous study (Nduka et al. 2019). At higher adsorbent dosages, the *q*_*t*_ decreases because the total amount of removable pollutants in the solution is fixed. As more adsorbents are added, the same quantity of contaminants becomes distributed among a greater number of adsorbents. Additionally, particle aggregation at higher dosages may reduce the effective surface area and hinder access to active sites, further contributing to the decline in adsorption capacity. (Chen et al. 2021c). Although the lowest dosage (0.5 g 50 mL^−1^) achieved an adsorption capacity of 1.86 mg g^−1^, the highest dosage (5 g 50 mL^−1^) yielded only 0.38 mg g^−1^.

**TABLE 3:** The *R* ^2^ value of the C33 sand kinetics model with various dosages added to 50 mL of wastewater.

First order	0.5 g	1.0 g	2.0 g	5.0 g
TSS	0.9887	0.9312	0.9427	0.9368
BOD	0.7879	0.8348	0.7998	0.8562
TN	0.9894	0.9892	0.9711	0.9759
TP	0.9085	0.9503	0.9578	0.9686
FC	0.9940	0.9845	0.9382	0.9628

Second order	0.5 g	1.0 g	2.0 g	5.0 g
TSS	0.9849	0.9774	0.9810	0.9654
BOD	0.9999	0.9997	0.9997	0.9999
TN	0.9303	0.9901	0.9328	0.9855
TP	0.9949	0.9935	0.9961	0.9988
FC	0.9600	0.9866	0.9966	0.9992

Abbreviations: BOD, biological oxygen demand; FC, fecal coliform; TN, total nitrogen; TP, total phosphorus; TSS, total suspended solids.

Th.

TN exhibited a similar trend to TSS, with a slower adsorption process requiring approximately 2 h to stabilize (Figure S5). An approximately sevenfold difference in adsorption capacity was observed between the lowest dosage (1.4633 mg g^−1^) and the highest dosage (0.2058 mg g^−1^). Inversely, TP adsorption was minimal and rapid, reaching completion within 20 min (Figure S6). These results aligned with the beaker test findings, which also indicated low phosphorus removal by C33 sand. Although other contaminants showed adsorption capacities of at least 1.4 mg g^−1^, TP was below 0.1 mg g^−1^ that was two orders of magnitude lower. The low adsorption capacity is probably the reason why the required reaction time is short because it is easy to attain its completion.

Overall, the *R* ^2^ value indicated that the pseudo‐second‐order kinetic model provided a better fit (Table 3) to the experimental data (*R* ^2^ ≥ 0.93) than the pseudo‐first‐order model (*R* ^2^ as low as 0.78), consistent with previous findings (Rauf et al. 2008). This suggests that the adsorption process involves chemisorption or other mechanisms beyond simple physisorption (Nduka et al. 2019). Additionally, the high *R* ^2^ values (≥ 0.99) between simulated and experimental *q*_*e*_ further support the feasibility of using kinetic models for *q*_*e*_ prediction (Figure S3–S7).

A kinetic study on the best‐performing biochar material (SP) with its optimal dosage (1 g 50 mL^−1^) was conducted, and the results are displayed in Figure S8–S12 and Table 4, with C33 sand at the same dosage included for comparison. For TSS (Figure S8), both SP and C33 sand exhibited similar trends, with uptake gradually increasing over time and reaching a maximum at approximately 2 h. SP removed more TSS (2.13 mg g^−1^) from septic effluent than C33 sand (1.13 mg g^−1^). Similarly, BOD removal followed comparable patterns for both materials (Figure S9). The reaction was faster compared to TSS adsorption and achieved its final status in an hour. At the same dosage, SP had the ability to adsorb 0.75 BOD mg g^−1^ more than C33 sand.

**TABLE 4:** The *R* ^2^ value of alternative materials kinetics model with their individual best dosage.

First order	SP	IES
TSS	0.9858	0.8201
BOD	0.8529	0.9455
TN	0.9555	0.9952
TP	0.9028	0.9731
FC	0.6652	0.9854

Second order	SP	IES
TSS	0.9700	0.9996
BOD	0.9999	0.7090
TN	0.9996	0.9987
TP	0.9625	0.9803
FC	1.0000	0.9963

Abbreviations: BOD, biological oxygen demand; FC, fecal coliform; IES, iron‐enhanced‐sand; SP, softwood pine; TN, total nitrogen; TP, total phosphorus; TSS, total suspended solids.

For TN and TP, adsorption increased rapidly at first and then slowed down at around 120 min and 40 min, respectively (Figure S10–11). TP clearly reached equilibrium faster than TN. Overall, SP's higher adsorption capacity suggests it could enhance the septic system's ability to reduce nutrient levels. The adsorption behavior for FC differed between SP and C33 sand (Figure S12). While SP reached equilibrium within 10 min, C33 sand was slower. However, the equilibrium adsorption capacities of the two materials were not significantly different (*p* value > 0.05, two‐tail student *t* test with *ɑ* = 0.05) (C33: 17,100 # g^−1^ and SP: 17,675 # g^−1^).

The *R* ^2^ values indicated that the pseudo‐second‐order kinetic model better described SP's adsorption behavior (Table 4), consistent with previous findings (Fan et al. 2017). An exception was observed for TSS, which fit the pseudo‐first‐order model slightly better, though the difference was not statistically significant. For adsorption capacity prediction (Table 5), the pseudo‐second‐order model demonstrated substantially higher accuracy. Most contaminants showed single‐digit errors under the second‐order model, whereas the first‐order model produced errors in the double to triple digits. It is clear from both simulation and experimental sides that using SP increases treatment capability across a range of pollutants.

**TABLE 5:** Prediction of softwood pine (SP) adsorption capacity at equilibrium using pseudo‐first and second‐order models.

First order	Actual *q*_*e*_ (mg g−1)	Simulated *q*_*e*_ (mg g−1)	Error (%)
TSS	2.13	2.27	6.29
BOD	4.75	2.21	−114.61
TN	1.45	0.95	−52.51
TP	0.33	0.46	28.92
FC^a^	17,625	2184	−707.19

Second order	Actual *q*_*e*_ (mg g^−1^)	Simulated *q*_*e*_ (mg g^−1^)	Error (%)
TSS	2.13	2.68	20.49
BOD	4.75	4.85	2.06
TN	1.45	1.50	3.56
TP	0.33	0.36	9.78
FC	17,625	16,66	−5.75

*Note:* Error (%) = (simulated − actual) / simulated.

Abbreviations: BOD, biological oxygen demand; FC, fecal coliform; TN, total nitrogen; TP, total phosphorus; TSS, total suspended solids.

^a^ The unit of *q*_*e*_ of fecal coliform is # g^−1^.

Unlike the other materials, IES adsorbed TSS rapidly, reaching equilibrium within 20 min (Figure S13), whereas SP and C33 sand required over an hour to stabilize. IES could remove more TSS (1.19 mg g^−1^) than C33 sand (0.85 mg g^−1^) under the same dosage in line with the beaker test results (Figure 1). In contrast, BOD removal by IES was slower (Figure S14). The adsorption capacity of IES (1.30 mg g^−1^) was approximately half that of C33 (2.15 mg g^−1^).

For TN, IES initially outperformed C33 sand up to 40 min (Figure S15). However, its adsorption rate slowed thereafter, whereas C33 sand continued to increase uptake throughout the 4‐h study. As observed in the beaker test, IES was not effective at removing TN, with a final adsorption capacity of only 0.25 mg g^−1^, which was 56.82% of C33's capacity (0.44 mg g^−1^). Conversely, IES demonstrated significantly higher adsorption capacity for TP, approximately 10 times greater than C33 sand (Figure S16). Although C33 sand reached equilibrium for TP within 20 min, IES continued adsorbing TP throughout the 4‐h period, achieving an adsorption capacity of ~0.2 mg g^−1^. For FC (Figure S17), C33 initially adsorbed more rapidly, but IES accelerated in the later phase and closed the gap. Ultimately, the difference in adsorption capacity between the two materials was not statistically significant (*p* value > 0.05, two‐tail student *t* test with ɑ = 0.05) (C33: 8187.5 # g^−1^ and IES: 8062.5 # g^−1^).

As with the other materials, the pseudo‐second‐order kinetic model provided a better fit for IES adsorption data, except for BOD (Table 4). The lower BOD removal efficiency suggests that physisorption may be the dominant mechanism, which aligns with the assumptions of the pseudo‐first‐order model. For all other contaminants, the pseudo‐second‐order model yielded *R* ^2^ values above 0.98, indicating strong agreement with experimental data. Simulated adsorption capacities (*q*_*e*_) from the pseudo‐second‐order model also showed lower average error percentages (Table 6), reinforcing its suitability for modeling adsorption kinetics across different materials.

**TABLE 6:** Prediction of iron‐enhanced‐sand (IES) adsorption capacity at equilibrium using pseudo‐first and second‐order models.

First order	Actual *q*_*e*_ (mg g^−1^)	Simulated *q*_*e*_ (mg g^−1^)	Error (%)
TSS	1.16	0.72	−60.41
BOD	1.28	1.51	15.65
TN	0.21	0.19	−10.97
TP	0.19	0.18	−8.56
FC^a^	8100	6534	−23.96

Second order	Actual *q*_*e*_ (mg g^−1^)	Simulated *q*_*e*_ (mg g^−1^)	Error (%)
TSS	1.16	1.20	3.72
BOD	1.28	2.41	47.15
TN	0.21	0.26	18.23
TP	0.19	0.23	16.77
FC	8100	10,000	19.00

*Note:* Error (%) = (simulated − actual) / simulated.

Abbreviations: BOD, biological oxygen demand; FC, fecal coliform; TN, total nitrogen; TP, total phosphorus; TSS, total suspended solids.

^a^ The unit of *q*_*e*_ of fecal coliform is # g^−1^.

### 3.3 Comparison and Implications

Table 7 provides a comparison between the results of this study and several prior investigations. The removal efficiency of TSS in the current study was notably lower than that reported in the referenced studies, likely due to methodological differences. Testing herein employed beaker tests, whereas all references listed in Table 7 used column tests, which will be the next step (Part II) of this alternative material research. In addition to all the reduction mechanisms that occur in beaker tests, column tests also achieve filtration due to their inherent design. The absence of filtration in beaker tests likely explains the lower TSS removal efficiencies reported in the present study.

**TABLE 7:** Removal efficiency (%) comparison between this study and published studies. Optimal results from this study are presented.

Material	Wastewater	TSS/turbidity	TOC/COD/BOD	Nitrogen	Phosphorus	Bacteria	Refs.
C33 Sand	Septic effluent	66.67	83.64	57.44	21.33	90.85	This study
Sand	Septic effluent	79.17–81.67	51.11–71.11	−2.36 to 31.13	24.77–45.87	81.67–98.19	Sauer David et al. (1976 )
Sand	Settled gray water	80–85	61–67	42–51	51	83.19–94.34	Katukiza et al. (2014 )
Sand	Settled gray water	79–86	61–64	39–43	49–52	82.52–89.12	Katukiza et al. (2014 )
IES	Septic effluent	82.22	46.36	23.53	95.51	91.27	This study
Iron	Gray water	96.1	46.3	43.7	—	98.8	Gupta et al. (2023 )
Iron	Gray water	—	59	56	82	98.9	Raj et al. (2023 )
Iron	Municipal	93	30.95	Negative	—	90	Verma et al. (2019 )
SP	Septic effluent	75.55	86.36	80.94	80.15	99.30	This study
Biochar	Gray water	95	63	76	—	—	Mwenge and Seodigeng (2019 )
Biochar	Municipal	89	90	64	78	—	Manyuchi et al. (2018 )
Biochar	Septage	61–83	86–91	—	—	97.04–99.98	de Rozari et al. (2015 )

Abbreviations: BOD, biological oxygen demand; COD, chemical oxygen demand; IES, iron‐enhanced‐sand; SP, softwood pine; TOC, total organic carbon; TSS, total suspended solids.

C33 sand demonstrated superior removal of organic matter and nitrogen but less effective phosphorus removal compared to commercial sand (Sauer David et al. 1976) and silica sand (Katukiza et al. 2014). Because both organic matter and nitrogen reduction require oxidation reactions, it is inferred that C33 sand creates a more oxidative environment. Nonetheless, it does not appear to support the chemical conditions necessary for effective phosphorus removal. In the current study, C33 sand achieved a 91% removal efficiency for bacteria, which falls within the range reported in the literature. Quantitative studies indicate that sand provides limited removal of nitrogen and phosphorus from septic effluent, nutrients that contribute to eutrophication in downstream aquatic ecosystems, with reported removal efficiencies ranging from −2% to 57% and 20% to 52%, respectively.

IES tested herein provided removal efficiencies similar to other studies for organic matter, nitrogen, and bacteria and superior removal efficiency for phosphorus. If improving phosphorus removal from septic effluent is a priority, then finding a cost‐effective method to treat septic effluent with IES appears to be a fruitful opportunity for future research.

On average, SP removal efficiency of TN, TP, and bacteria was superior to prior published studies. As noted earlier, the beaker test methodology used in this study does not capture the filtration benefits present in column tests or in situ drainfields. Therefore, it is anticipated that removal efficiencies could be even higher in future experiments using column setups that better simulate real‐world conditions.

Based on the results presented here, successful incorporation of SP and IES into conventional septic systems could significantly reduce downstream nitrogen and phosphorus loading to groundwater and surface waters. Assuming (i) 1 trillion gallons of septic effluent are discharged annually in the USA (EPA 2002), (ii) septic effluent quality is equivalent to septic effluent characterized in Table 2, (iii) all soil treatment areas currently use C33 sand, and (iv) SP and IES treatment could be universally implemented, then nationwide water quality improvements could reach approximately 3.66E + 04 tons TSS, 1.25E + 04 tons BOD, 3.51E + 04 tons nitrogen, 2.51E + 04 tons phosphorus, and 1.25E + 08 CFU FC. Furthermore, the current prices of SP (Char 2025) and IES (Connelly‐GPM 2025) are only $0.11 and $0.025 USD more per pound, respectively, than C33 sand (Quarry 2025). For a typical three‐bedroom single‐family home, incorporating SP and IES could potentially reduce overall costs, as their optimal dosages are 5 and 2.5 times lower, respectively, than that of C33 sand on a per‐weight basis.

## 4 Conclusions and Future Research

This study identified optimal materials and dosages for improving septic effluent treatment. For C33, the best‐performing dosage was 5 g 50 mL^−1^, whereas the highest dosage (10 g 50 mL^−1^) would lead to negative TSS removal efficiency, likely due to fine particle interference. In the biochar screening test, most biochars disintegrated into the water, which resulted in negative TSS removal efficiency, an undesirable outcome for septic system applications. However, SP was selected as the ideal candidate that provided sufficient material integrity to avoid disintegration while achieving promising removal efficiencies for TSS, BOD, TP, TN, and FC. IES was chosen for its optimal performance in removing TSS and TP. Dosage tests revealed comparable results between 2 and 5 g 50 mL^−1^, so 2 g 50 mL^−1^ was selected based on cost‐effectiveness; that is, 2‐g IES is 60% less expensive than 5‐g IES. If C33 sand were replaced with SP and IES at their optimal dosages, pollutant removal efficiencies would likely improve as follows: TSS from 67% to 82%, BOD from 84% to 86%, TN from 57% to 81%, TP from 21% to 96%, and FC from 91% to 99%. Additionally, the cost for constructing soil treatment areas would likely be reduced.

Future research will be based on the results of Part I. The best‐performing materials and dosages will be applied in column systems to determine optimal packing configuration and assess long‐term performance. The durability results will also be used to compare the adsorption amount at equilibrium in this manuscript. Furthermore, the effectiveness of this new drainfield design in removing emerging contaminants will be evaluated, expanding the scope of its environmental benefits.

## Author Contributions

**Chia‐Yang Chen:** data curation, formal analysis, investigation, methodology, validation, visualization, writing – original draft. **Sara Heger:** conceptualization, funding acquisition, project administration, supervision, writing – review and editing. **D. Albrey Arrington:** software, supervision, visualization, writing – review and editing. **Bo Hu:** resources, supervision, writing – review and editing.

## Conflicts of Interest

The authors declare no conflicts of interest.

## Supporting information


**Table S1:** The literature review of household wastewater treatment using a septic system with sand filtration.
**Table S2:** Different materials with various dosages were added into flask containing septic effluent in this study. Quantified wastewater quality parameters of supernatant included total suspended solids (TSS), biological oxygen demand (BOD), total nitrogen (TN), total phosphorus (TP), and fecal coliform (FC).
**Figure S1:** Particle size distribution of various materials. Bar represents the weight percentage of that mesh layer while line is the accumulated percentage. (IES‐Iron‐enhanced‐sand, ZVI‐Zero‐Valent iron, IT‐Iron tailings, BD‐Biochar DG, BP‐Biochar Pure, BA‐Black ash, RP‐Red pine, SP‐Softwood pine, SC‐Softwood chunk, NC‐Naked char, and HP‐Hardwood powder).
**Figure S2:** Photos taken during experimentation show notable observations. (a) Flasks with 50‐mL septic effluent plus 5‐g (left) and 10‐g (right) C33 sand. Note the cloudy water in the flask containing 10‐g C33 sand (right), which supports the inference of fine and friable particles that contributed to increased TSS. (b) Flasks with 50‐mL septic effluent plus 5‐g biochars showing biochar disintegration. (c) Flasks with 50‐mL septic effluent plus 5‐g iron products showing ferric oxide formation.
**Figure S3:** C33 kinetics experiment with various dosage and pseudo‐first/second‐order model on total suspended solids (TSS) (Dot: experimental value; Line: simulated value).
**Figure S4:** C33 kinetics experiment with various dosage and pseudo‐first/second‐order model on biological oxygen demand (BOD) (Dot: experimental value; Line: simulated value).
**Figure S5:** C33 kinetics experiment with various dosage and pseudo‐first/second‐order model on total nitrogen (TN) (Dot: experimental value; Line: simulated value).
**Figure S6:** C33 kinetics experiment with various dosage and pseudo‐first/second‐order model on total phosphorus (TP) (Dot: experimental value; Line: simulated value).
**Figure S7:** C33 kinetics experiment with various dosage and pseudo‐first/second‐order model on fecal coliform (Dot: experimental value; Line: simulated value).
**Figure S8:** The kinetic study of Softwood pine (SP) with the best dosage (1 g 50 mL^−1^) on total suspended solids (TSS) (Dot: experimental value; Line: simulated value).
**Figure S9:** The kinetic study of Softwood pine (SP) with the best dosage (1 g 50 mL^−1^) on biological oxygen demand (BOD) (Dot: experimental value; Line: simulated value).
**Figure S10:** The kinetic study of Softwood pine (SP) with the best dosage (1 g 50 mL^−1^) on total nitrogen (TN) (Dot: experimental value; Line: simulated value).
**Figure S11:** The kinetic study of Softwood pine (SP) with the best dosage (1 g 50 mL^−1^) on total phosphorus (TP) (Dot: experimental value; Line: simulated value).
**Figure S12:** The kinetic study of Softwood pine (SP) with the best dosage (1 g 50 mL^−1^) on fecal coliform (Dot: experimental value; Line: simulated value).
**Figure S13:** The kinetic study of Iron‐enhanced‐sand (IES) with the best dosage (2 g 50 mL^−1^) on total suspended solids (TSS) (Dot: experimental value; Line: simulated value).
**Figure S14:** The kinetic study of Iron‐enhanced‐sand (IES) with the best dosage (2 g 50 mL^−1^) on biological oxygen demand (BOD) (Dot: experimental value; Line: simulated value).
**Figure S15:** The kinetic study of Iron‐enhanced‐sand (IES) with the best dosage (2 g 50 mL^−1^) on total nitrogen (TN) (Dot: experimental value; Line: simulated value).
**Figure S16:** The kinetic study of Iron‐enhanced‐sand (IES) with the best dosage (2 g 50 mL^−1^) on total phosphorus (TP) (Dot: experimental value; Line: simulated value).
**Figure S17:** The kinetic study of Iron‐enhanced‐sand (IES) with the best dosage (2 g 50 mL^−1^) on Fecal Coliform (Dot: experimental value; Line: simulated value).

## Data Availability

The data that support the findings of this study are available from the corresponding author upon reasonable request.
