# Supplementary material for: Evaluation of Biochar and Iron as Alternative Materials to Improve Performance of Septic Systems—Part 1. Material Selection and Batch Tests
Source: Water Environ Res. 2025 Nov 26;97(12):e70207. doi: 10.1002/wer.70207 (PMC12658315; doi:10.1002/wer.70207)
Supplement: Supplementary file 1 — Table S1: The literature review of household wastewater treatment using a septic system with sand filtration. Table S2: Different materials with various dosages were added into flask containing septic effluent in this study. Quantified wastewater quality parameters of supernatant included total suspended solids (TSS), biological oxygen demand (BOD), total nitrogen (TN), total phosphorus (TP), and fecal coliform (FC). Figure S1: Particle size distribution of various materials. Bar represents the weight percentage of that mesh layer while line is the accumulated percentage. (IES‐Iron‐enhanced‐sand, ZVI‐Zero‐Valent iron, IT‐Iron tailings, BD‐Biochar DG, BP‐Biochar Pure, BA‐Black ash, RP‐Red pine, SP‐Softwood pine, SC‐Softwood chunk, NC‐Naked char, and HP‐Hardwood powder). Figure S2: Photos taken during experimentation show notable observations. (a) Flasks with 50‐mL septic effluent plus 5‐g (left) and 10‐g (right) C33 sand. Note the cloudy water in the flask containing 10‐g C33 sand (right), which supports the inference of fine and friable particles that contributed to increased TSS. (b) Flasks with 50‐mL septic effluent plus 5‐g biochars showing biochar disintegration. (c) Flasks with 50‐mL septic effluent plus 5‐g iron products showing ferric oxide formation. Figure S3: C33 kinetics experiment with various dosage and pseudo‐first/second‐order model on total suspended solids (TSS) (Dot: experimental value; Line: simulated value). Figure S4: C33 kinetics experiment with various dosage and pseudo‐first/second‐order model on biological oxygen demand (BOD) (Dot: experimental value; Line: simulated value). Figure S5: C33 kinetics experiment with various dosage and pseudo‐first/second‐order model on total nitrogen (TN) (Dot: experimental value; Line: simulated value). Figure S6: C33 kinetics experiment with various dosage and pseudo‐first/second‐order model on total phosphorus (TP) (Dot: experimental value; Line: simulated value). Figure S7: C33 kinetics experiment with var [file WER-97-e70207-s001.docx]

Supporting Information

**Evaluation of biochar and iron as alternative materials to improve performance of septic systems – Part 1. Material selection and batch tests**

Chia-Yang Chen ^1,2*^, Sara Heger ^1^, D. Albrey Arrington ^2^, Bo Hu ^3^

1. Water Resources Center, University of Minnesota, 1985 Buford Avenue, Saint Paul, MN 55108, USA
2. Loxahatchee River District, 2500 Jupiter Park Drive, Jupiter, FL 33458, USA
3. Department of Bioproducts and Biosystems Engineering, University of Minnesota, 1390 Eckles Ave, Saint Paul, MN 55108, USA

* Corresponding author. *E-mails*: [CY.Chen@lrecd.org](mailto:CY.Chen@lrecd.org); [jyc81512@gmail.com](mailto:jyc81512@gmail.com)

Number of Figures: 17

Number of Table: 2

Number of Pages: 24

**Table S1**

The literature review of household wastewater treatment using a septic system with sand filtration.

| Sand | Important properties | Operating condition | Wastewater  (mg/L) | Outcomes | Ref. |
| --- | --- | --- | --- | --- | --- |
| Commercialized Sand | **Effective size**  0.45 mm  **Sand Depth**  24 inches  **Uniformity**  3  **Surface area**  13.75 ft^2^ | **Intermittent**  0.56-1.68 m/d  (Trial 1)  0.2 m/d  (Trial 2)  8-25 times/d (Trial 1)  4-13 times/d  (Trial 2) | **Septic tank effluent**  BOD: 120  COD: 289  TSS: 45  VSS: 33  NH_4_: 20.9  NOx: 0.3  PO_4_: 10.9  Fecal Coli.: 5.4 E+5 numbers/100 ml  Total Coli.: 2.0 E+6 numbers/100 ml | After sand filtration  BOD: 22-25  COD: 77-85  TSS: 13-22  VSS: 7-9  NH_4_: 13.6-16.0  NOx: 1.0-5.7  PO_4_: 5.9-8.2  Fecal Coli.: 2.7-9.8 E+3 numbers/100 ml  Total Coli.: 1.0-2.3 E+4 numbers/100 ml | **(Sauer David, Otis Richard, & Boyle William, 1976)** |
| Sand | **Effective size**  0.44 mm  **Sand Depth**  12-24 inches  **Uniformity**  3.3  **Surface area**  2.58 ft^2^ | **Intermittent**  0.236 m/d  (Trial 1)  0.255 m/d  (Trial 2)  3.7 L/min (Trial 1)  7.6 L/min (Trial 2) | **Septic tank effluent**  (with minor ammonia additive)  BOD: 115 (Trial 1)  BOD: 161 (Trial 2)  TSS: 56 (Trial 1)  TSS: 75 (Trial 2)  NH_4_: 25 (Trial 1)  NH_4_: 25 (Trial 2) | After sand filtration  BOD: 12-28 (Trial 1)  BOD: 20-40 (Trial 2)  TSS: 10-16 (Trial 1)  TSS: 16-28 (Trial 2)  NH_4_: 0.53-3.6 (Trial 1)  NH_4_: 0.39-3.0 (Trial 2) | **(L. Widrig, A. Peeples, & M. Mancl, 1996)** |
| Coarse sand  Fine sand  Glass sand | **Effective size**  0.52 mm (Coarse)  0.27 mm (Fine)  0.20 mm (Glass)  **Sand Depth**  10 inches (each) | **Intermittent**  1.5 gal/d/sq ft | **Septic tank effluent**  Enteroviruses  1.6 E+3 to 3.3 E+7  PFU/L | After sand filtration  Enteroviruses  0 to 6.2 E+3  PFU/L | **(Gross Mark & Mitchell, 1990)** |
| River sand | **Size**  0.80-1.18 mm (Sand 1)  0.40-0.80 mm (Sand 2)  1.18-4.75 mm (Coarse)  4.75-12.00 mm (Gravel)  **Sand Depth**  5cm  (Gravel and Coarse)  40 cm  (Each sand) | **Intermittent**  20 L/day | **Coagulated greywater**  BOD: 39  COD: 80  Turbidity: 12 NTU  EC: 815 µS/cm  pH: 7.04  Fecal Coli.: 1.7 E+4  MPN/100 ml  Total Coli.: 2.1 E+5  MPN/100 ml | After filtration  BOD: 21-27  COD: 40-47  Turbidity: 4-5 NTU  EC: 898-883 µS/cm  pH: 7.51-7.62  Fecal Coli.: 2.0-3.7 E+2  MPN/100 ml  Total Coli.: 5.3-9.1 E+3  MPN/100 ml | **(Singh, Ahammed, & Shaikh, 2021)** |
| Sand | **Sand Depth**  15 cm | **Continuous**  4 m/h  4 hours/cycle | **Coagulated greywater**  COD: 163  TSS: 37  pH: 4.85 | After sand filtration  COD: 156  TSS: 22  pH: 5.91 | **(Antonopoulou, Kirkou, & Stasinakis, 2013)** |
| Silica sand  Crushed lava rock | **Size**  0.5-2.56 mm (Crushed lava rock)  0.5-2.56 mm (Silica Sand)  **Effective size**  0.95 mm  (Crushed lava rock)  0.65 mm  (Silica Sand)  **Uniformity**  1.89  (Crushed lava rock)  4.00  (Silica Sand)  **Surface area**  3.1 m^2^/g  (Crushed lava rock)  0.43 m^2^/g  (Silica Sand)  **Sand Depth**  15 cm  (Gravel)  60 cm  (Crushed lava rock for column 1)  30cm+30cm  (Crushed lava rock + silica sand for column 2) | **Constant**  20 cm/d  40 cm/d | **Settled greywater**  pH: 7.2  DO: 2.2  BOD: 1125  COD: 2861  TOC: 892  DOC: 559  TSS: 996  NH_4_: 24.7  NOx: 3.8  TKN: 58.5  TP: 2.9  PO_4_: 2.7  E. Coli: 4.2 E+6 numbers/100 ml  Total Coli.: 6.9 E+7 numbers/100 ml | After filtration (removal efficiency)  Column 1  BOD: 61-67  COD: 70  TOC: 66.7-71  DOC: 65-69  TSS: 80-85  NH_4_: 62-69  NOx: 54-55  TKN: 42-51  TP: 51  PO_4_: 48-51  E. Coli: 2.31-2.52 log removal  Total Coli.: 1.62-1.83 log removal  Column 2  BOD: 61-64  COD: 69-70  TOC: 69-70  DOC: 61-66.8  TSS: 79-86  NH_4_: 61-68  NOx: 51-54  TKN: 39-43  TP: 49-52  PO_4_: 48  E. Coli: 1.98-2.18 log removal  Total Coli.: 1.55-1.65 log removal | **(Katukiza, Ronteltap, Niwagaba, Kansiime, & Lens, 2014)** |
| Sand | **Effective size**  1.4 mm  (Sand)  **Uniformity**  2.2  (Sand)  **Surface area**  0.14 m^2^/g  (Sand)  **Sand Depth**  60 cm  (Sand)  **Porosity**  34% | **Intermittent**  360 cm/hour  (32-128 L/m^2^/day)  3 times/day  70/10/20% of total daily load | **Synthetic greywater**  pH: 6.98-8.28  BOD: 126-2390  COD: 420-4800  NH_4_: 4.1-47.3  NOx: 1.6-7.8  TN: 25-241  TP: 0.73-10.23  PO_4_: 0.51-10.3 | After sand filtration  (Removal efficiency)  BOD: 73-93  COD: 65-86  TN: 3  TP: 22-85 | **(Dalahmeh et al., 2014)** |
| River sand | **Effective size**  0.41 mm  **Uniformity**  5.85  **Sand Depth**  25 cm  (Fine sand)  5 cm  (Each for very fine, fine, and coarse gravels) | **Intermittent**  Hydraulic loading  0.56, 0.85, 1.12 cm/hour  Idle time  18 hours | **Sewage**  pH: 7.15  Turbidity: 34  TSS: 208  COD: 162.78  NOx: 2.91  PO_4_: 2.29  Fecal Coli.: 6-1600  MPN/100 ml  Total Coli.:15-8000  MPN/100 ml | After sand filtration  (Removal efficiency)  Turbidity: 93.10-94.33  TSS: 65.68-87.36  COD: 47.9-56.53  NOx: Negative  PO_4_: Negative-35.94  Fecal Coli.: 96.99->99  Total Coli.: 90.41->99 | **(Verma, Daverey, & Sharma, 2019)** |

**Table S2**

Different materials with various dosages were added into flask containing septic effluent in this study. Quantified wastewater quality parameters of supernatant included total suspended solids (TSS), biological oxygen demand (BOD), total nitrogen (TN), total phosphorus (TP), and fecal coliform (FC).

| Batch test | Purpose | Material | Dosage (g)/50 ml | Exposure Duration (minutes) |
| --- | --- | --- | --- | --- |
| 1^st^ | Identify optimal dose of C33 sand | C33 sand | 0.5, 1.0, 2.0, 5.0, and 10.0 | 1440 |
| 2^nd^ | Identify optimal biochar type | Eight biochar materials | The best result from 1^st^ (5.0) | 1440 |
| 3^rd^ | Identify optimal iron product | Three iron products | The best result from 1^st^ (5.0) | 1440 |
| 4^th^ | Optimize biochar dosage | The best result from 2^nd^ (SP) | 0.5, 1.0, 2.0, 5.0, and 10.0 | 1440 |
| 5^th^ | Optimize iron dosage | The best result from 3^rd^ (IES) | 0.5, 1.0, 2.0, 5.0, and 10.0 | 1440 |
| Kinetic test | Purpose | Material | Dosage (g)/50 mL | Exposure Duration (minutes) |
| 1^st^ | Kinetics model simulation | C33 sand | 0.5, 1.0, 2.0, and 5.0^+^ | 5, 10, 20, 40, 60, 90, 120, and 240^*^ |
| 2^nd^ | Kinetics model simulation | The best result from 2^nd^ (SP) | The best result from 4^th^ (1.0) | 5, 10, 20, 40, 60, 90, 120, and 240 |
| 3^rd^ | Kinetics model simulation | The best result from 3^rd^ (IES) | The best result from 5^th^ (2.0) | 5, 10, 20, 40, 60, 90, 120, and 240 |

^+^Because of the volume of sample size needed for eight samples, all materials (C33 sand, SP, IES, and septic effluent) were proportionally increased by 20 times. (SP-Softwood pine and IES-Iron-enhanced-sand)

^*^The kinetic test was stopped at 240 minutes because the adsorption capacity at that time was within a 3% error range of the capacity at 1440 minutes (or within 0.05 mg g^-1^ if the adsorption capacity was less than 0.5 mg g^-1^)


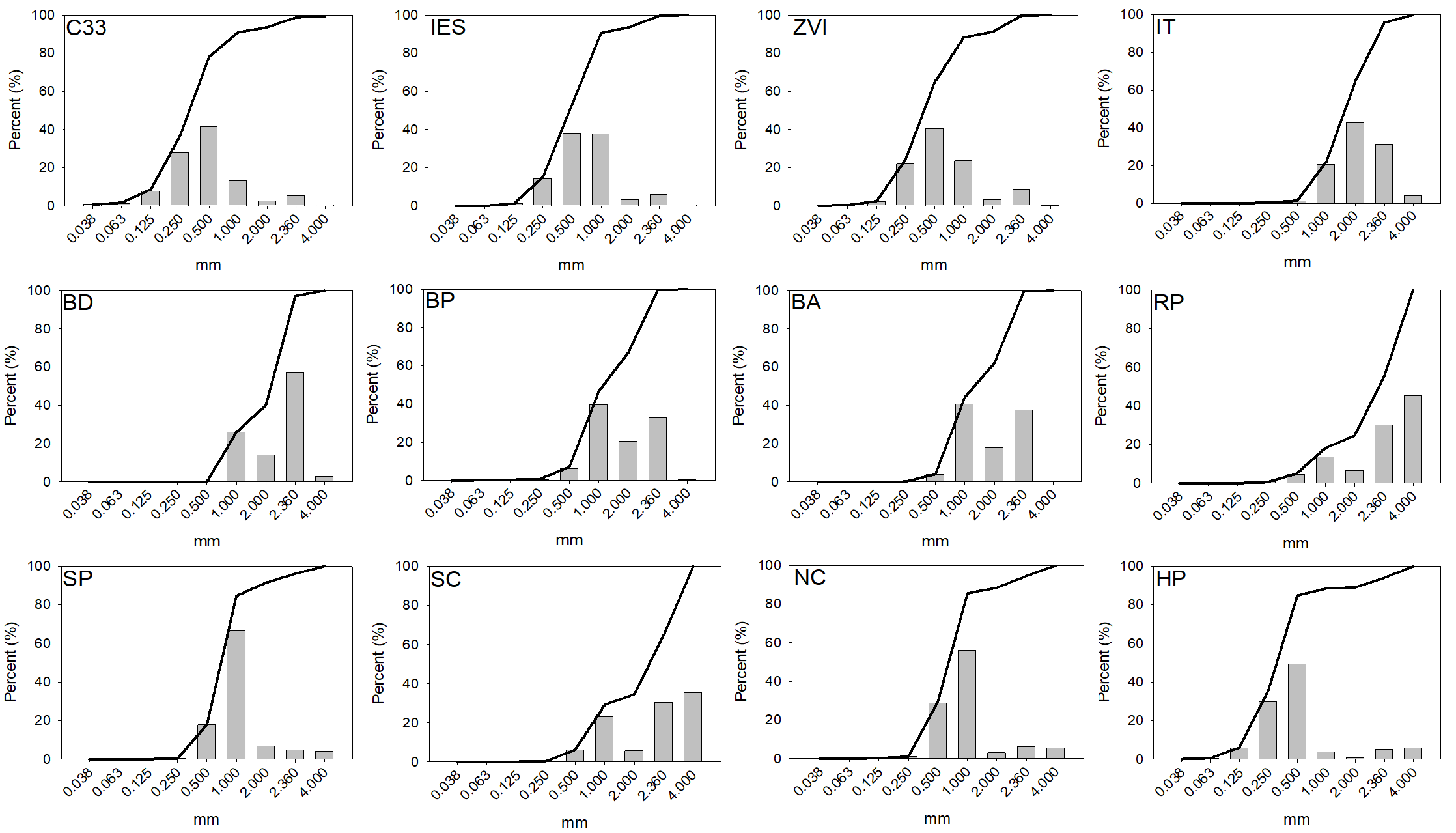


**Fig. S1** Particle size distribution of various materials. Bar represents the weight percentage of that mesh layer while line is the accumulated percentage. (IES-Iron-enhanced-sand, ZVI-Zero-Valent iron, IT-Iron tailings, BD-Biochar DG, BP-Biochar Pure, BA-Black ash, RP-Red pine, SP-Softwood pine, SC-Softwood chunk, NC-Naked char, and HP-Hardwood powder).


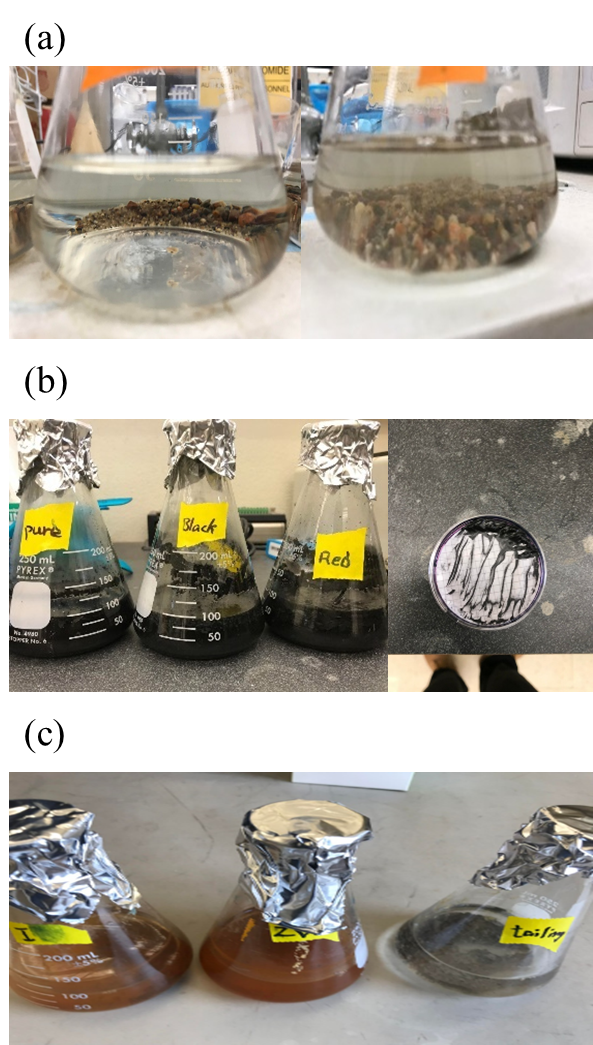


**Fig. S2** Photos taken during experimentation show notable observations. (a) Flasks with 50 ml septic effluent plus 5 g (left) and 10 g (right) C33 sand. Note the cloudy water in the flask containing 10 g C33 sand (right), which supports the inference of fine and friable particles that contributed to increased TSS. (b) Flasks with 50 ml septic effluent plus 5 g biochars showing biochar disintegration. (c) Flasks with 50 ml septic effluent plus 5 g iron products showing ferric oxide formation.





**Fig. S3** C33 kinetics experiment with various dosage and pseudo-first/second-order model on total suspended solids (TSS) (Dot: experimental value; Line: simulated value).

**Fig. S4** C33 kinetics experiment with various dosage and pseudo-first/second-order model on biological oxygen demand (BOD) (Dot: experimental value; Line: simulated value).

**Fig. S5** C33 kinetics experiment with various dosage and pseudo-first/second-order model on total nitrogen (TN) (Dot: experimental value; Line: simulated value).

**Fig. S6** C33 kinetics experiment with various dosage and pseudo-first/second-order model on total phosphorus (TP) (Dot: experimental value; Line: simulated value).


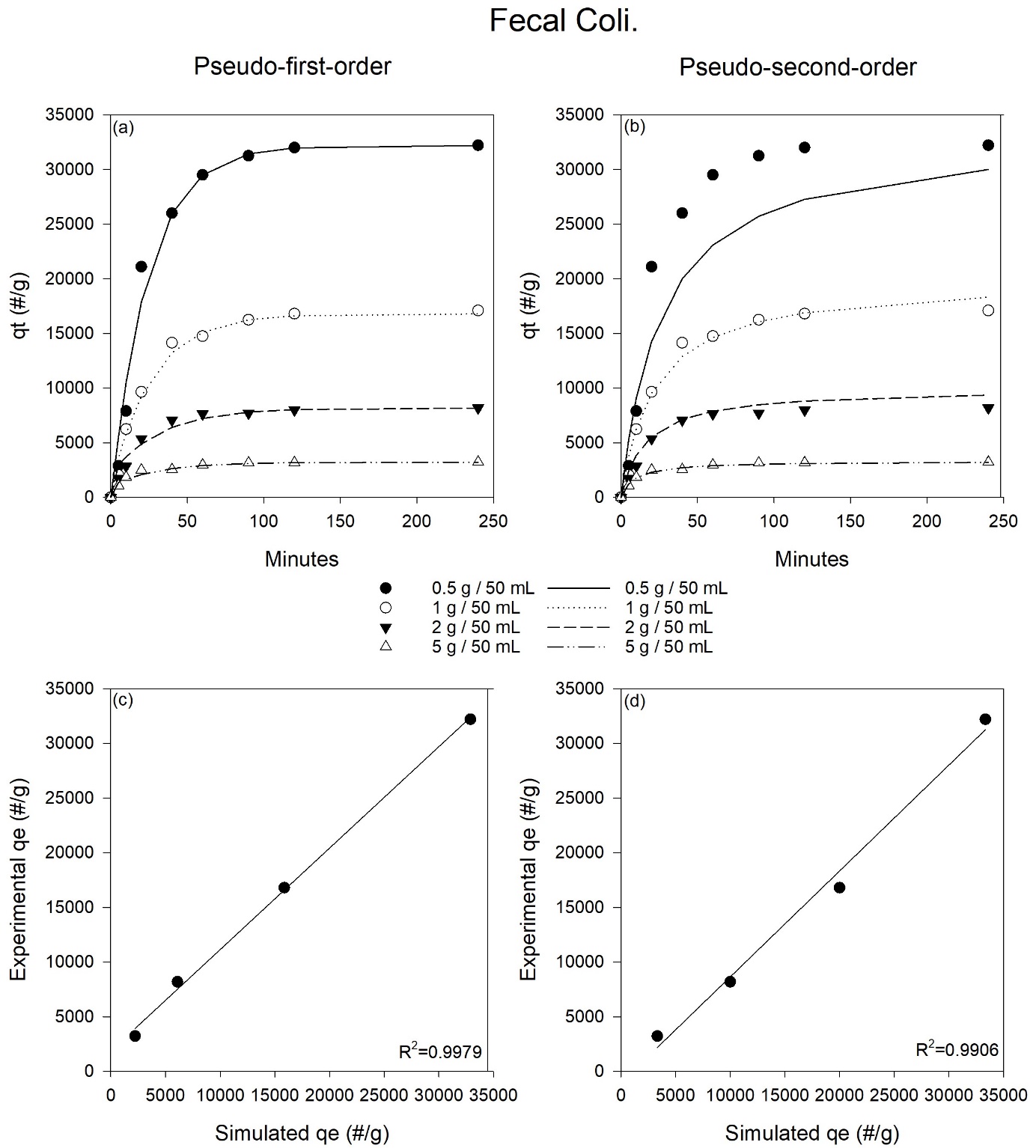


**Fig. S7** C33 kinetics experiment with various dosage and pseudo-first/second-order model on fecal coliform (Dot: experimental value; Line: simulated value).

**Fig. S8** The kinetic study of Softwood pine (SP) with the best dosage (1 g 50 ml^-1^) on total suspended solids (TSS) (Dot: experimental value; Line: simulated value).

**Fig. S9** The kinetic study of Softwood pine (SP) with the best dosage (1 g 50 ml^-1^) on biological oxygen demand (BOD) (Dot: experimental value; Line: simulated value).

**Fig. S10** The kinetic study of Softwood pine (SP) with the best dosage (1 g 50 ml^-1^) on total nitrogen (TN) (Dot: experimental value; Line: simulated value).

**Fig. S11** The kinetic study of Softwood pine (SP) with the best dosage (1 g 50 ml^-1^) on total phosphorus (TP) (Dot: experimental value; Line: simulated value).

**Fig. S12** The kinetic study of Softwood pine (SP) with the best dosage (1 g 50 ml^-1^) on fecal coliform (Dot: experimental value; Line: simulated value).

**Fig. S13** The kinetic study of Iron-enhanced-sand (IES) with the best dosage (2 g 50 ml^-1^) on total suspended solids (TSS) (Dot: experimental value; Line: simulated value).

**Fig. S14** The kinetic study of Iron-enhanced-sand (IES) with the best dosage (2 g 50 ml^-1^) on biological oxygen demand (BOD) (Dot: experimental value; Line: simulated value).

**Fig. S15** The kinetic study of Iron-enhanced-sand (IES) with the best dosage (2 g 50 ml^-1^) on total nitrogen (TN) (Dot: experimental value; Line: simulated value).

**Fig. S16** The kinetic study of Iron-enhanced-sand (IES) with the best dosage (2 g 50 ml^-1^) on total phosphorus (TP) (Dot: experimental value; Line: simulated value).

**Fig. S17** The kinetic study of Iron-enhanced-sand (IES) with the best dosage (2 g 50 ml^-1^) on Fecal Coliform (Dot: experimental value; Line: simulated value).

Reference

Antonopoulou, G., Kirkou, A., & Stasinakis, A. S. (2013). Quantitative and qualitative greywater characterization in Greek households and investigation of their treatment using physicochemical methods. *Science of The Total Environment, 454-455*, 426-432. doi:<https://doi.org/10.1016/j.scitotenv.2013.03.045>

Dalahmeh, S. S., Pell, M., Hylander, L. D., Lalander, C., Vinnerås, B., & Jönsson, H. (2014). Effects of changing hydraulic and organic loading rates on pollutant reduction in bark, charcoal and sand filters treating greywater. *Journal of Environmental Management, 132*, 338-345. doi:<https://doi.org/10.1016/j.jenvman.2013.11.005>

Gross Mark, A., & Mitchell, D. (1990). Virus Removal by Sand Filtration of Septic Tank Effluent. *Journal of Environmental Engineering, 116*(4), 711-720. doi:10.1061/(ASCE)0733-9372(1990)116:4(711)

Katukiza, A. Y., Ronteltap, M., Niwagaba, C. B., Kansiime, F., & Lens, P. N. L. (2014). Grey water treatment in urban slums by a filtration system: Optimisation of the filtration medium. *Journal of Environmental Management, 146*, 131-141. doi:<https://doi.org/10.1016/j.jenvman.2014.07.033>

L. Widrig, D., A. Peeples, J., & M. Mancl, K. (1996). Intermittent Sand Filtration for Domestic Wastewater Treatment: Effects of Filter Depth and Hydraulic Parameters. *Applied Engineering in Agriculture, 12*(4), 451-459. doi:<https://doi.org/10.13031/2013.25670>

Sauer David, K., Otis Richard, J., & Boyle William, C. (1976). Intermittent Sand Filtration of Household Wastewater. *Journal of the Environmental Engineering Division, 102*(4), 789-803. doi:10.1061/JEEGAV.0000513

Singh, S., Ahammed, M. M., & Shaikh, I. N. (2021). Combined coagulation and intermittent sand filtration for on-site treatment of greywater. *IOP Conference Series: Materials Science and Engineering, 1114*(1), 012031. doi:10.1088/1757-899x/1114/1/012031

Verma, S., Daverey, A., & Sharma, A. (2019). Wastewater treatment by slow sand filters using uncoated and iron-coated fine sand: impact of hydraulic loading rate and media depth. *Environmental Science and Pollution Research, 26*(33), 34148-34156. doi:10.1007/s11356-018-3551-4
